# A Medicinal Chemistry Perspective on Excitatory Amino Acid Transporter 2 Dysfunction in Neurodegenerative Diseases

**DOI:** 10.1021/acs.jmedchem.2c01572

**Published:** 2023-02-14

**Authors:** Igor C. Fontana, Débora G. Souza, Diogo O. Souza, Antony Gee, Eduardo R. Zimmer, Salvatore Bongarzone

**Affiliations:** 1 School of Biomedical Engineering and Imaging Sciences, St Thomas’ Hospital, King’s College London, London SE1 7EH, United Kingdom; 2 Graduate Program in Biological Sciences: Biochemistry, Universidade Federal do Rio Grande do Sul, Rua Ramiro Barcelos, 2600, 90035-003 Porto Alegre, Brazil; 3 Division of Clinical Geriatrics, Center for Alzheimer Research, Department of Neurobiology, Care Sciences and Society, Karolinska Institutet, Blickagången 16 - Neo floor seventh, 141 83 Stockholm, Sweden; 4 Department of Biochemistry, Universidade Federal do Rio Grande do Sul, Rua Ramiro Barcelos, 2600, 90035-003 Porto Alegre, Brazil; 5 Department of Pharmacology, Universidade Federal do Rio Grande do Sul, Av. Sarmento Leite 500, sala, 90035-003 Porto Alegre, Brazil; 6 Graduate Program in Biological Sciences: Biochemistry (PPGBioq), and Pharmacology and Therapeutics (PPGFT), Universidade Federal do Rio Grande do Sul, Av. Sarmento Leite 500, sala, 305 Porto Alegre, Brazil; 7 Brain Institute of Rio Grande do Sul, Pontifical Catholic University of Rio Grande do Sul, Av. Ipiranga, 6681 Porto Alegre, Brazil; 8 McGill University Research Centre for Studies in Aging, McGill University, Montreal, Quebec H4H 1R3, Canada

## Abstract

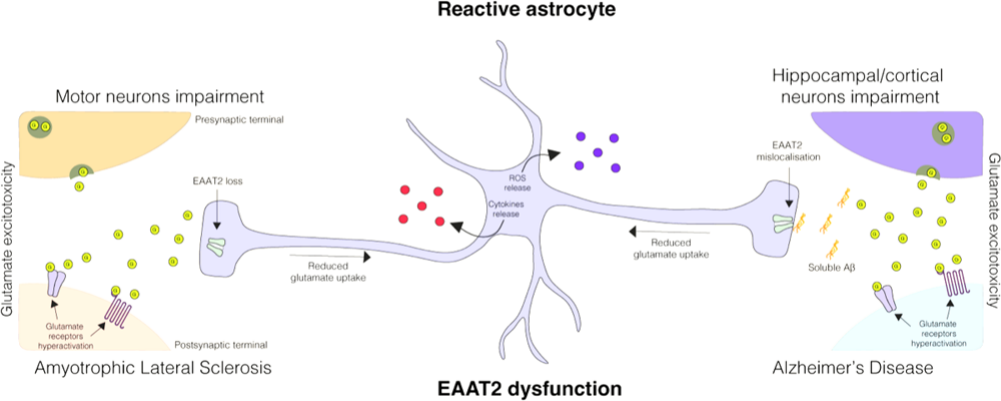

The excitatory amino acid transporter 2 (EAAT2) plays a key role in the clearance and recycling of glutamate - the major excitatory neurotransmitter in the mammalian brain. EAAT2 loss/dysfunction triggers a cascade of neurodegenerative events, comprising glutamatergic excitotoxicity and neuronal death. Nevertheless, our current knowledge regarding EAAT2 in neurodegenerative diseases, such as amyotrophic lateral sclerosis (ALS) and Alzheimer’s disease (AD), is restricted to post-mortem analysis of brain tissue and experimental models. Thus, detecting EAAT2 in the living human brain might be crucial to improve diagnosis/therapy for ALS and AD. This perspective article describes the role of EAAT2 in physio/pathological processes and provides a structure–activity relationship of EAAT2-binders, bringing two perspectives: therapy (activators) and diagnosis (molecular imaging tools).

## 1 Introduction

**Figure 1:**
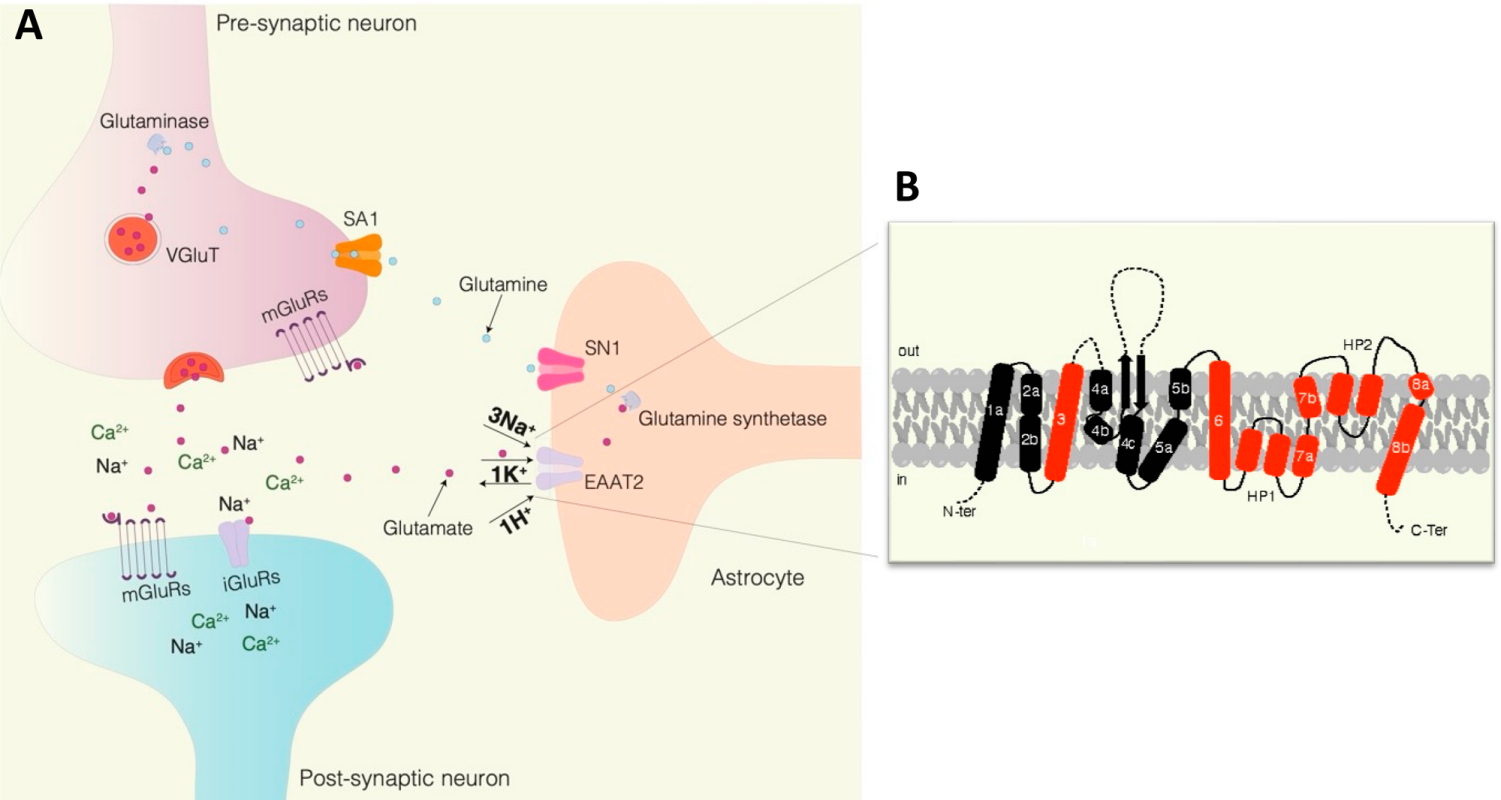
Glutamate-glutamine cycle in the healthy brain. (A) Astrocytes are known to provide the glutamine required by neurons to synthesize GABA and glutamate. The glutamine efflux from the astrocytes is mediated via the system N transporter (SN1). Once in the extracellular space, neurons capture glutamine through the system A transporters (SA1 and SA2). In the neuronal intracellular space, glutamine is metabolized into glutamate by the mitochondrial enzyme glutaminase. Glutamate is then packed within synaptic glutamatergic vesicles (VGluT) via a Mg^2+^/ATP-dependent process and released to the extracellular compartment from the vesicles by a Ca^2+^-dependent mechanism. Of note, the glutamate concentration in the intracellular and extracellular space is 10 mM and 10 μM, respectively. Once glutamate is released in the synaptic cleft, it produces an excitatory postsynaptic potential, which is tightly controlled by a wide range of neuronal receptors, including ionotropic and metabotropic glutamate receptors (iGluRs and mGluRs). A balance between glutamate release and clearance is essential. Glutamate in the extracellular space is captured by astrocytes via EAAT2. (B) Topology diagram of EAAT2. The transport domain consists TM3, TM6, TM7, TM8, HP1, HP2 and the connecting HP1 and HP2 loops.

Glutamate, the primary excitatory neurotransmitter in the mammalian brain, is a poorly blood-brain barrier (BBB) penetrant nonessential amino acid, requiring synthesis within the central nervous system (CNS).^1−3^ In the CNS, glutamate is mostly synthesized via two classical pathways. The first is a *de novo* synthetic route using glucose and the anaplerotic enzyme pyruvate carboxylase, yielding glutamate by further transamination of α-ketoglutarate.^4,5^ The second is the glutamine-glutamate cycle which involves the exchange of these two amino acids between neurons and astrocytes (Figure 1).^6^ Astrocytes cease glutamatergic neurotransmission by removing glutamate from the synaptic cleft via the excitatory amino acid transporter 2 (EAAT2, human isoform) or the glutamate transporter 1 (GLT-1, rodent isoform that shares 96% identity with the human EAAT2).^7−9^

The reduced capacity of removing extracellular glutamate by EAAT2—due to transporter mislocalization^10^ or degradation^11^—is associated with a pathological phenomenon termed glutamatergic excitotoxicity, a common feature in neurodegenerative diseases such as Alzheimer’s disease (AD), amyotrophic lateral sclerosis (ALS), Parkinson’s disease (PD), and Huntington’s disease (HD).^12−15^ Experimental models and post-mortem analysis of human brain tissue from patients with AD, ALS, PD, and HD suggest that EAAT2 loss/dysfunction could be an early trigger evolving into chronic reactive astrogliosis, oxidative stress, and neuronal death.^16−18^ In this context, identifying molecules able to increase EAAT2 levels (increasing EAAT2 expression through translational activation or preventing EAAT2 degradation) or enhance EAAT2 function (positive allosteric modulator, PAM, promoting glutamate binding) could lead the way for the development of therapeutic agents, while EAAT2 binders (inhibitors and PAM) could inspire the design of novel imaging diagnostic tools.

Minimal invasive neuroimaging techniques, such as positron emission tomography (PET), are key to evaluate neurotransmitter trafficking and transporter density. Although the use of PET radiolabeled glutamate (e.g., [^11^C]glutamate) may work to investigate neurotransmitter trafficking and availability of its transporters/receptors, it would lack of selectivity and potentially be converted to glutamine, confounding imaging results.^19,20^ Note that there are no brain-penetrant EAAT2-selective PET radiotracers available for clinical research (an early phase 1 EAAT2 targeted positron emitting agent, [^18^F]fluorenylasparaginate methyl ester - [^18^F]RP-115 - started to recruit on May 2022 for which no published data are available yet, clinical trials: NCT05374378).

The initial step for developing a PET radiotracer targeting EAAT2 should be the selection of a molecule with high affinity and selectivity toward EAAT2. Therefore, in this perspective article, we conduct a structure–activity relationship of the library of EAAT2 inhibitors and PAMs defining appropriate chemical features that might help with the development of EAAT2 selective PET radiotracers. In addition, we discuss important findings regarding EAAT2 structure/isoforms and examine experimental evidence of EAAT2 dysfunction/loss in neurodegenerative diseases to emphasize the importance of detecting EAAT2 *in vivo*.

## 2 A Snapshot of the Glutamatergic Neurotransmission and Transporters

The brain is formed by a complex and intertwined network of cells that communicate in a subsecond frequency. Excitatory neurotransmission is largely—more than 90%—accounted by the glutamatergic system.^2,21,22^ Glutamate, once released by neurons, binds to a group of high-affinity ionotropic receptors (NMDAr , KAr and AMPAr) and eight isoform metabotropic glutamatergic receptors (e.g., mGluR_1–8_),^23−25^ until eventually most of the extracellular glutamate is captured by EAAT’s.^26,27^ There are five known EAATs in the mammalian body: EAAT1 (GLAST for rodents),^28^ EAAT2 (GLT-1 for rodents),^29^ EAAT3 (EAAC1 for rodents),^29^ EAAT4,^30^ and EAAT5.^31^ Immunohistochemical analyses suggested that EAAT1 and EAAT2 are mainly localized in the membrane of astrocytes in the CNS.^32,33^ EAAT3 is predominantly found in the soma and dendrites of both excitatory and inhibitory neurons as well as EAAT4 is largely expressed in Purkinje cells and in the membrane of postsynaptic neurons.^34,35^ EAAT5 is exclusively located in the retinal ganglion cells.^36^

### 2.1 Excitatory Amino Acid Transporter 2

The EAAT2, a membrane-bound protein composed of 574 amino acids, is responsible for more than 95% of total glutamate uptake in the forebrain region of the adult mammalian brain^37^ (steady state affinity (*K*_m_) of 10–20 μM and binding affinity (*K*_d_) of 140 μM).^38^ Importantly, more than 80% of total EAAT2 expression in the mammalian brain is detected in the membrane of astrocytes.^39^ Glutamate transport via EAAT2 is accompanied by the entry of three sodium ions and one proton, combined with the release of one potassium ion (Figure 1).^28^ EAAT2 has eight α-helical transmembrane regions (TMs 1–8) and two helical hairpin loops (HP1 and HP2).^40^ The EAAT2 usually forms a homotrimer to enable glutamate uptake, in which TMs 2–5 are responsible for trimerization of EAAT2.^41^ The transport domain consists of four TMs (TM3, TM6, TM7, and TM8), HP1, HP2 and the connecting HP1 and HP2 loops, respectively.

### 2.2 EAAT2 Splicing and Localization

The EAAT2 belongs to the solute carrier 1 (SLC1) family encoded by the SLC1A2 gene. A single functional nucleotide polymorphism, rs4354668 (181 T/G), located in the gene promoter region seems to modulate the expression of EAAT2.^28^ Indeed, EAAT2 has a complex pattern of alternative splicing mostly at the C-terminal position. The main splice variant of EAAT2 in the human brain is termed EAAT2b (99.5% identity to the full-length EAAT2), which uniquely differs in the C-terminal amino acid sequence.^42^ The full-length EAAT2 represents more than 90% of total EAAT2 content in the forebrain region (25-fold higher than EAAT2b in the whole brain).^43,44^ EAAT2 is mainly expressed in the caudate nucleus, nucleus basalis of Meynert, spinal ventral horn, cerebral cortex, and hippocampus and found in lower levels in other CNS regions.^45^ In addition, expression of EAAT2 (GLT-1) in rat hippocampus and cerebellum presents 12,000 and 2800 molecules per μm^3^ tissue, respectively.^46^

### 2.3 Binding Sites

**Figure 2:**
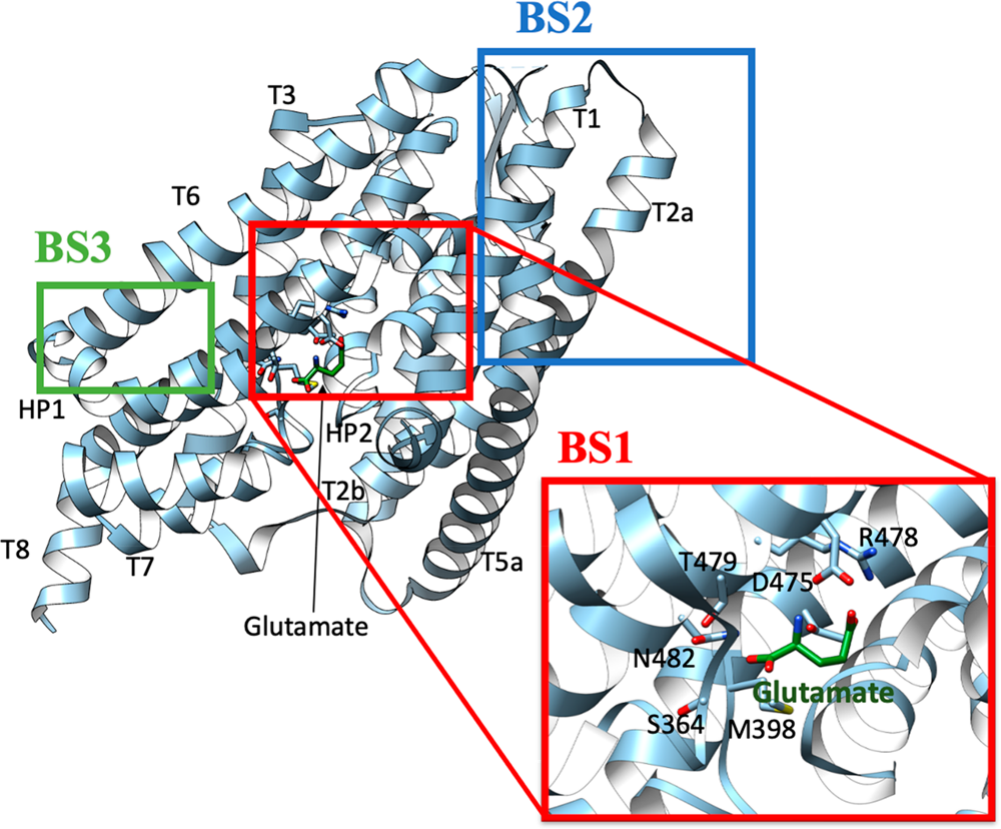
Three-dimensional structure of EAAT2 in complex with glutamate (PDB code 7XR4). Close-ups on the glutamate binding site (BS1). Two peripherical binding sites (BS2 and BS3) are also shown.

A crystal structure analysis of the glutamate transporter of *Pyrococcus horikoshii* (Glt_Ph_)—a bacterial homologue that shares 37% identity with human EAAT2—helped build our knowledge about EAAT2 binding pockets.^40^ To date, one binding site (BS1) for substrates (glutamate/aspartate) and two binding sites (BS2 and BS3) for modulators have been identified (Figure 2). BS1 is formed in the C-terminal portion of each EAAT2 monomer, which includes HP1, HP2, TM7, and TM8 (Figures 1 and 2). In fact, the D475 and R478 amino acid residues in TM8 are suggested to be the main players in the formation of this binding pocket.^47−49^ Two independent research groups have releveled the cryo-electron microscopy (cryo-EM) structural information on human EAAT2 in complex at BS1 with glutamate^50^ (Figure 2) and a selective inhibitor WAY-213613.^50,51^ BS2 is formed by the H71 amino acid residue in the TM2, L295 and K299 in TM5, and W472 in TM8 (Figure 2).^52^ More recently, BS3 defined by M477 on TM8, and F345, F348, F352, W355 on HP1 was identified as a binding pocket for negative allosteric modulators (Figure 2).^51,53^

## 3 EAAT2 in Brain Disorders

Over the last decades, EAAT2 loss/dysfunction in neurodegenerative diseases became a potential target for pharmacological intervention in the brain. Here, we briefly discuss experimental findings and immunohistochemical reports of post-mortem human brain tissue which suggest that EAAT2 impairment and glutamatergic excitotoxicity precede neuronal loss and clinical manifestations in ALS and AD and other neurodegenerative diseases.

### 3.1 Amyotrophic Lateral Sclerosis

ALS is a progressive adult-onset neurodegenerative disease affecting neurons associated with voluntary muscle movement.^54^ ALS is the most common motor neuron disease in adults, and only 5–10% of cases have a genetic link—familial ALS (fALS).^55^ In fALS mutated superoxide dismutase 1 (SOD1)—a critical enzyme for maintaining cellular redox homeostasis—adopts an aberrant conformation causing protein aggregation.^56^ The common ALS clinical symptoms are fasciculations, tight muscles, difficulty chewing, and muscle cramps.^57^ Such clinical manifestations are primarily due to selective degeneration of upper and lower motor neurons, which are located in the motor cortex and in the brainstem and spinal cord, characterizing the main CNS regions affected in this disease.^58^ Even though ALS etiology remains unknown, glutamate excitotoxicity and EAAT2 impairment could be potential triggers in ALS development.^59^ Interestingly, sporadic ALS patients have considerable loss in EAAT2 protein levels in the motor cortex (71 ± 12%) and spinal cord (57 ± 12%) compared to healthy subjects.^14^ In addition, it seems that EAAT2 loss could originate from aberrant mRNA processing.^60^ Specifically, brain regions most affected by ALS pathology presented reduced levels of EAAT2 mRNA variants that retained intron-7 or skipped exon-9 (EAAT2-e9).^60,61^ Further work could not confirm that the reduction of this EAAT2 translational variant was specific of ALS, thus whether or not the intron-7 and the exon-9 variants are differentially expressed in ALS patients compared to healthy subjects remains under debate.^62,63^

A transgenic mouse model bearing the Cu^2+^/Zn^2+^ SOD1 mutation was developed to explore ALS pathophysiology.^64,65^ Interestingly, in SOD1 mice the EAAT2 loss of function and expression preceded neuronal loss and disease onset.^66^ Of note, disturbances on glutamatergic neurotransmission are associated with disease progression and seem to be region-specific, mostly affecting the spinal cord.^67^*In vitro* studies corroborate these results, showing hypertrophy of glial fibrillary acidic protein (GFAP)-positive astrocytes (also an index of reactive astrogliosis) and alterations in EAAT2 levels prior to motor neuron loss.^68^ Reactive astrocytes in ALS are known to mediate the release of inflammatory cytokines such as tumor necrosis factor-α (TNF-α).^69^ TNF-α and downstream NFκB signaling have been previously shown to suppress EAAT2 expression.^70^ Interestingly, it seems that deletion of membralin, an endoplasmic reticulum protein, in SOD-1 mice is a key figure in this process. More specifically, membralin deletion exacerbated the activation of TNF-α receptor and NFκB pathway and reduced EAAT2 transcription.^71^

Gibb and colleagues investigated if EAAT2 splicing could be involved in the EAAT2 dysfunction in ALS.^72^ Using the SOD-1 mouse model, the authors observed that EAAT2 impairment could be linked to caspase-3 activation.^72^ Caspase-3 cleaved EAAT2 at the cytosolic C-terminal domain producing two fragments: (1) truncated EAAT2 and (2) carboxy terminus of EAAT2 (CTE). Then, CTE is SUMOylated (CTE-SUMO-1) and accumulates in the spinal cord of SOD-1 mice.^72^ CTE-SUMO-1 accumulation was mostly seen in the presymptomatic stage and specific mouse brain regions related to ALS.^72^ In agreement with these findings, a mutation on the EAAT2 site for caspase-3 cleavage extended mice life span and delayed the progression of motor changes.^73^

### 3.2 Alzheimer’s Disease

Over the last decades, EAAT2 loss/dysfunction in AD, the most common cause of dementia, became a potential target for pharmacological intervention. Here, we briefly discuss immunohistochemical reports from post-mortem human brains and experimental models showing that EAAT2 dysfunction/loss and glutamatergic excitotoxicity may precede neurodegeneration and clinical manifestations in the AD continuum.

AD is characterized by a slow decline in memory, thinking, and reasoning abilities, mainly occurring in its sporadic form. Genetic-linked AD represents less than 1% of the cases (mostly related to mutations in the genes of amyloid precursor protein, presenilin 1 and 2).^74^ The clinical manifestation of AD includes memory loss, space/time disorientation, and behavioral symptoms such as depression and personality changes.^75^ AD pathogenesis, according to the National Institute of Aging – Alzheimer’s Association research framework, is defined by a biological construct (based on fluid and imaging biomarkers) comprising Aβ deposition (A), pathologic tau (T) and neurodegeneration (N) – the AT(N) system.^76^ Remarkably, biological changes in AD patients are suggested to start 20–30 years before the symptomatic stages; therefore, the need for identifying new biomarkers to detect these early alterations is fundamental to improving disease diagnosis and therapy.^77^ In this context, glial cells (mostly astrocytes and microglia) are currently suggested to be early responders to pathological changes in AD.^78−80^ Astrocytes undergo functional, morphological, and molecular changes in response to AD pathology—a phenomenon termed reactive astrogliosis. Early evidence showing that astrocytes become reactive in AD was mostly associated with upregulated mRNA and protein levels of the GFAP.^81,82^ Nevertheless, the current view on reactive astrogliosis indicates that a single biomarker, such as GFAP upregulation, is unlikely to represent the whole picture of reactive astrocytes in the human brain.^83^ For instance, decreased expression level of EAAT2 characterizes an additional biomarker to identify reactive astrocytes in AD.^83^ The several lines of research on EAAT2 in AD are discussed below and subdivided in human brain findings and animal/cellular models of AD.

### 3.3 Immunohistochemical Reports of Post-Mortem Human AD Brain Tissue

Immunohistochemical reports of post-mortem AD brain tissue indicated that EAAT2 density loss correlates with upregulated levels of GFAP in the temporal cortex of AD individuals, a region of high Aβ pathology.^84^ In a recent meta-analysis of post-mortem immunohistochemical reports, EAAT2 was downregulated in AD versus control brains.^85^ Although lower expression of EAAT2 in AD brains is not a consensus,^86,87^ the presence of EAAT2 mRNA variants could also account for these inconsistencies observed by different groups.^88^ Specifically, higher expression of two exon-skipping mRNA variants (EAAT2-e7 and EAAT2-e7e9) was observed in brain regions commonly affected by AD pathology, while wild-type EAAT2 mRNA levels were decreased in post-mortem brain tissue of AD patients.^88^ Remarkably, the increase of mRNA variants of EAAT2-e7 and EAAT2-e7e9 seems to correlate with pathology severity in these brains.^88^ Furthermore, reactive astrocytes are very prone to oxidative stress, and, EAAT2, in particular, stands out as a specific target of reactive oxygen species in astrocytes.^89^ Woltjer et al. demonstrated that detergent insoluble EAAT2—possibly derived from oxidation processes—accumulates in the hippocampus and frontal cortex of AD patients and correlates with disease progression.^90^ In keeping with this, post-translational modifications by either defective mRNA splicing or oxidative damage may affect the availability of EAAT2 in the cell surface, reducing the glutamate uptake capacity and ultimately causing neuronal death.

### 3.4 Animal Models of Aβ Pathology: Immunohistochemistry Findings

Animal and cellular models are useful tools to further explore potential mechanisms that may explain the interplay between Aβ pathology, reactive astrogliosis, and EAAT2 loss.^91^ Different animal models seek to reproduce the main pathological hallmarks of AD. However, it is important to emphasize that rodent models such as APP/PS1 (mice containing human transgenes for APP Swedish mutation and PSEN1 containing an L166P mutation)^92^ or 5xFAD (mice containing 5 human AD-linked mutations) do not reflect the complexity of AD pathogenesis, but rather mimic amyloidosis in AD. Below, we discuss the main findings regarding EAAT2 loss/dysfunction in different models of Aβ pathology.

One of the first reports to evaluate EAAT’s levels in animal models of Aβ pathology, using transgenic mouse overexpressing human APP bearing the London mutation, demonstrated that decreased EAAT2 expression precedes the formation of insoluble Aβ aggregates in the neocortex, while EAAT2 mRNA levels remain stable.^93^ Similarly, Schallier and colleagues, using AβPP23 mice, showed that lower expression levels of EAAT2 in the pre-Aβ plaque stage occurs independently of GFAP upregulation in the frontal cortex and hippocampus.^94^ Furthermore, in APP/PS1 mice, partial loss of EAAT2 (genetically modified mice lacking one EAAT2 allele) accelerated memory impairment onset^95^ and associated with insoluble Aβ deposits.^96^ In a similar extent, lower levels of hippocampal EAAT2 and accelerated cognitive deficits were also observed in adeno-associated virus-based APP/PS1 mice.^97^ Nonetheless, it is important to mention that in the medial prefrontal cortex of 3xTg-AD mouse, EAAT2 levels were comparable to the wild-type animals.^98^

In summary, we speculate that functional changes (i.e., EAAT2 loss and decreased glutamate uptake capacity) may precede astrocytic morphological alterations (e.g., GFAP increased levels) and deposition of insoluble Aβ aggregates in the early stages of AD. In addition, alterations in EAAT2 levels, which were not observed in all brain regions, suggest that EAAT2 loss is not a universal marker for reactive astrogliosis (i.e., some astrocytic populations may maintain EAAT2 levels unaltered), thus, corroborating with the concept of astrocyte heterogeneity in AD.

### 3.5 *In Vitro* Insights on Aβ-EAAT2 Interaction and Glutamatergic Excitotoxicity

Studies *in vitro* (cell culture and brain slices) offer the possibility of exploring mechanistic insights into the association between preplaque Aβ species (e.g., soluble Aβ oligomers, AβOs) and EAAT2 loss in astrocytes. In primary culture of astrocytes, soluble AβOs internalize EAAT2 in the astrocytic membrane disturbing glutamate uptake.^99^ Studies on brain hippocampal slices showed that blocking glutamate uptake by EAATs, reduced synaptic response via AMPAr, induced activation of extrasynaptic NMDA receptors and depolarization of astrocytic membrane.^100^ Other mechanisms have been proposed to explain EAAT2 loss in the membrane of astrocytes in the context of Aβ pathology, such as EAAT2 interaction with presenilin 1 (PS1)—the catalytic component of the amyloid precursor protein-processing enzyme, γ-secretase—and cholesterol metabolism.^101,102^ Specifically, increased cholesterol 24S-hydroxylase activity caused the reduction of cholesterol levels in the astrocytic membrane and led to the dissociation of EAAT2 from lipid rafts and, consequently, EAAT2 loss.^102^

Altogether, these findings highlight the strong association between initial Aβ pathology and EAAT2 dysfunction in AD, either in a direct manner (AβOs-induced decrease on EAAT2 levels by physical interaction and internalization of EAAT2 in the membrane of astrocytes or via downstream signaling pathways, e.g., CN/NFAT pathway^103^) or by alternative mechanisms associated with Aβ aggregation, such as cholesterol dyshomeostasis (a catalyzer of Aβ aggregation process^104^) and PS1 expression (associated with APP cleavage and Aβ formation).

### 3.6 Tau and EAAT2 Dysfunction

Disturbances in EAAT expression/function are tightly associated with early AD changes, including reactive astrogliosis and Aβ pathology, extensively discussed above. However, a link between pathological tau (usually viewed as a later pathological event in the AD continuum) and EAAT2 was demonstrated in temporal cortex homogenates from AD brains.^105^ Specifically, EAAT2 was shown to interact with hyperphosphorylated tau rather than nonpathological tau.^105^ In a transgenic mouse model of astrocytic tau pathology (GFAP/tau Tg mice), decreased EAAT2 levels were evident throughout the spinal cord and reflected on motor impairment in these animals. To a lesser extent, EAAT2 loss was observed in the occipital and frontal cortices.^106^ Complimentarily, in a very recent work it was shown that tau oligomers, following internalization into the astrocytic intracellular space, inhibited EAAT2 expression and decreased the capacity of astrocytes to uptake glutamate.^107^ These findings suggest that, perhaps, EAAT2 dysfunction and reactive astrogliosis are not only associated with Aβ pathology in AD but also with pathological tau in the later stages of the AD continuum.

### 3.7 Other Neurodegenerative Diseases

The implication of EAAT2 dysfunction/loss in other common neurodegenerative disease, such as HD and PD, has also been investigated, although not as much as compared to studies in AD and ALS brains and experimental models.

HD is a neurodegenerative disease characterized by cognitive, motor, and psychiatric dysfunction. The pathophysiology of HD is typically associated with an inherited gene—huntingtin—which disrupts multiple cellular processes leading to neuronal loss in basal ganglia and cortical regions of the human brain.^108^ Consistent evidence, based on immunohistochemical analysis of post-mortem HD brain tissue, indicated prominent loss of EAAT2 expression and mRNA levels in subcortical regions of the human brain such as striatum, neostriatum, and putamen.^109,110^ Interestingly, Arzberger and colleagues found that global EAAT2 mRNA levels decreased in neostriatum of HD, in correlation to disease severity, but identified increased number of astrocytes expressing EAAT2, putting forward a compensatory astrocytic mechanism to prevent glutamate excitotoxicity and neuronal death.^110^

PD is the second most prevalent neurodegenerative disease in which clinical manifestation, in the context of motor function impairment, overlaps with HD comprising bradykinesia, tremor and postural instability. PD pathophysiology is associated with misfolding of α-synuclein—a protein whose aggregation leads to the formation of inclusions termed Lewy bodies—and the progressive loss of dopaminergic neurons in the substantia nigra (a component of the basal ganglia). The involvement of EAAT2 was mostly demonstrated in experimental models of PD, highlighting the strong association between reactive astrocytes and EAAT2 loss in the striatum.^111−114^ Interestingly, Wnt1 promoted increased EAAT2 expression in astrocytes which induced protective effects on dopaminergic neurons.^115^ Another study reported that exercise intervention in 6-hydroxydopamine-induced PD rats significantly improved the motor dysfunction of PD model rats, increased the ability of striatal glutamate reuptake significantly, and upregulated the expression levels of EAAT2 protein.^116^ In human brains, a recent work showed that EAAT2 trafficking in the astrocytic membrane is affected by the leucine-rich repeat kinase 2 (LRRK2) pathogenic variant G2019S, which is considered a contributor of late onset familial PD.^117^ Specifically, EAAT2 downregulation in the striatum correlated with increased GFAP positive astrocytes in caudate and putamen of PD patients as compared to healthy controls.^117^ Research in the context of reactive astrogliosis in PD pathogenesis is prominent.^118,119^ Therefore, detecting EAAT2 *in vivo* could be an additional tool to increase our knowledge on the role of reactive astrocytes in the early stages of PD.

## 4 EAAT2 Activators

The involvement of EAAT2 loss/dysfunction in neurodegenerative diseases suggests that restoring its expression/function could be an alternative to halt the downstream cascade of neurodegenerative processes or, at least, to attenuate the excitotoxic process. In this context, transcriptional (increase gene transcription) and translational (increase the protein expression) activators and/or reducing protein turnover can increase EAAT2 expression, while PAMs restore the EAAT2 function.^18^ Therapeutic targeting EAAT2 seems promising, since higher EAAT2 expression was associated with improved cognitive function and brain energetic metabolism.^120,121^ In the paragraphs below, we discuss the whole library of EAAT2 activators and PAMs.

### 4.1 EAAT2 Transcriptional Activators

The beta-lactam antibiotic ceftriaxone increases EAAT2 expression at the transcriptional level through NF-κB-mediated EAAT2 promoter activation and restored EAAT2 function in animal models of ALS, AD, and PD by delaying loss of neurons and muscle strength, improving cognitive performance, and increasing survival.^96,122−125^ The clinical efficacy of ceftriaxone was tested so far in ALS,^126,127^ and PD (on going, NCT03413384). However, Fumagalli et al. showed that ceftriaxone was unable to prevent loss of EAAT2 levels and activity following growth factor withdrawal in primary mouse striatal astrocyte.^128^ Dexamethasone has been previously shown to be an efficient inducer of EAAT2 in cortical astrocytes and increase in activity in cortical and striatal astrocytes.^129,130^ Tamoxifen and Raloxifene increased EAAT2 protein expression by the activation of multiple signaling pathways including ERK, EGFR, and CREB mediated by estrogen receptors (ERs) ER-α, ER-β, and GPR30 as well as increased EAAT2 mRNA in astrocytes.^131−133^ Nevertheless, since mRNA levels of EAAT2 might not be affected in AD brains,^87,134^ transcriptional activators may not be the strategy to pursue for therapeutic intervention.

### 4.2 EAAT2 Translational Activators

**Figure 3:**
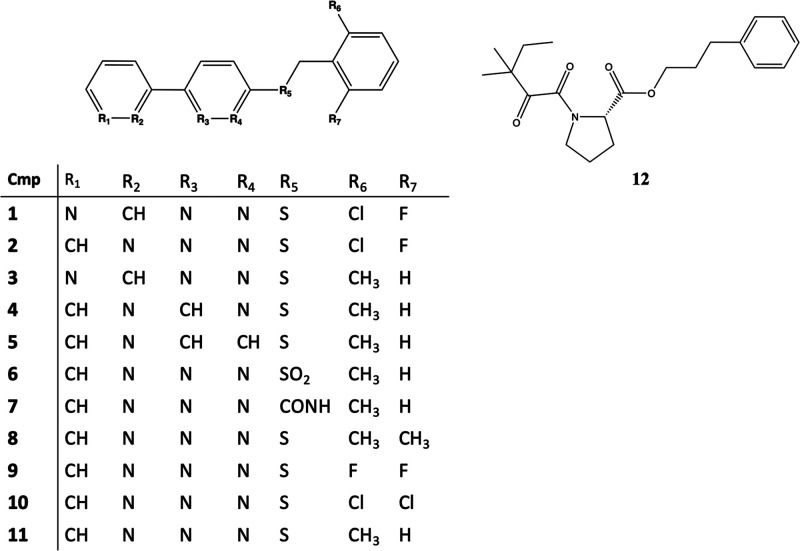
EAAT2 translational activators. Compound **11** is also known as LDN/OSU-0212320.

An initial high-throughput screen of 140,000 compounds identified translational activators of EAAT2 such as compound **1** (Figure 3), which increases EAAT2 protein levels 2-fold.^135^ Further exploitation of pyridazine-based compounds may help in determining the specific molecular components required for enhancing EAAT2 protein levels. The increase in EAAT2 protein levels compared to vehicle (dimethyl sulfoxide only) were adopted to estimate the potency of each compound. At first, it was demonstrated that replacing the 2-pyridyl in **1** for a 3-pyridyl (**2**) had a similar effect in increasing EAAT2 levels. Importantly, modifications in the pyridazine ring had a negative impact.^136^ Instead replacing the 2-Cl-6-F-Bn (**1**) with a 2-Me-Bn (**3**) or an unsubstituted Bn group, the EAAT2 level increased >3-fold. By removing one (**4**) or two nitrogen atoms (**5**) of the pyridazine ring, the increase on EAAT2 protein levels is completely lost for **4** or reduced by half compared to compound **1**, respectively.^136^ Furthermore, the sulfur link of **1** seems crucial for increasing EAAT2 levels, as oxidation into a Sulphone (**6**) or replacement to an amide group (**7**) decreased activity from 2-fold to 1.1- and 1.4-fold, respectively.^136^ The addition of a further methyl group on the aromatic ring of the benzyl group resulted in stronger activity as observed by comparing compounds bearing a 2,6-Di-Me-Bn (6.5-fold increase, **8**) to compound **1** (3.5-fold increase).^136^ Interestingly, the substitution of the two methyl groups in **8** with two fluorine atoms (**9**) or two chlorine atoms (**10**) decreases the effect from 6.5-fold to 2.2-fold or 3.9-fold, respectively. To further explore the efficacy of these pyridazine-based compounds, in a mixed culture of astrocytes and neurons, compound **11** (LDN/OSU-0212320) prevented glutamate excitotoxicity by selective increase on EAAT2 expression.^137^ In keeping with this, translational activators of EAAT2 may have a potential therapeutic application in neurodegenerative diseases in which EAAT2 damage occurs mostly at the posttranslational level, such as AD.^88,138^ Loss of EAAT2 protein in APP_Sw,Ind_ mice is caused by disturbances at the post-transcriptional level because EAAT2 mRNA is not decreased. The treatment with **11** of APP_Sw_, animal model of AD, reversed memory and learning deficits after a short period of treatment, sustained beneficial effects on cognitive functions even after 1 month of treatment cessation, indicating a potential for disease modification, restored synaptic integrity, and increased EAAT2 expression via the translational rather than the transcriptional activation mechanism, resolving the central problem of reduced EAAT2 expression.^139^ Other translational activators are sulbactam, amitriptyline, riluzole, GPI-1046, and MS-153. Sulbactam upregulated EAAT2 expression in rats and prevented or reversed the EAAT2 downregulation normally induced in the ischemic rat brain.^140,141^ Chronic amitriptyline administration to rats produced upregulation of EAAT1 and EAAT2 in spinal cord.^142^ Riluzole, an FDA-approved benzothiazole drug, may exert its neuroprotective effects by changing the relative affinity for glutamate to EAAT2^128^ and increasing EAAT2 levels.^129^ Moreover, Liu et al. showed that riluzole increased EAAT2 protein expression *in vitro*, a phenomenon associated with the increased expression of HSP70 and HSP90.^143^ The non-immunosuppressant neuroimmunophilin GPI-1046 (**12**) exerted both neuroprotective and neuroregenerative effects in cell culture and in animal models of ALS. **12** induced both expression and activity of EAAT2 in rat spinal cord cultures and in rat brain homogenates without appreciable effects on gene expression. Chronic oral administration of **12** prolonged survival in transgenic mouse model of ALS and produced increased levels of spinal cord EAAT2 protein.^144^ MS-153 enhanced the function of EAAT2 *in vitro*, and it has shown neuroprotective effects in multiple experimental models associated with glutamate excitotoxicity (including traumatic brain injury, ischemia, addiction, and anxiety).^145−149^ Nevertheless, it seems that MS-153 activity is mostly linked to modulation of calcium channel currents via interaction with protein kinase C, and not through EAAT2 activation.^150^

### 4.3 EAAT2 PAM

Activators/enhancers of EAAT2 activity could restore glutamate uptake and prevent glutamatergic excitotoxicity.^151^ Multiple attempts have been made over the last decades to design selective EAAT2 activators/enhancers.^152^ An example is Parawixin1, a compound isolated from the *Parawixia bistriata* spider, which enhanced EAAT2-mediated glutamate uptake by interacting with the trimerization region, located between TM2 and TM5 of EAAT2.^153,154^

**Figure 4:**
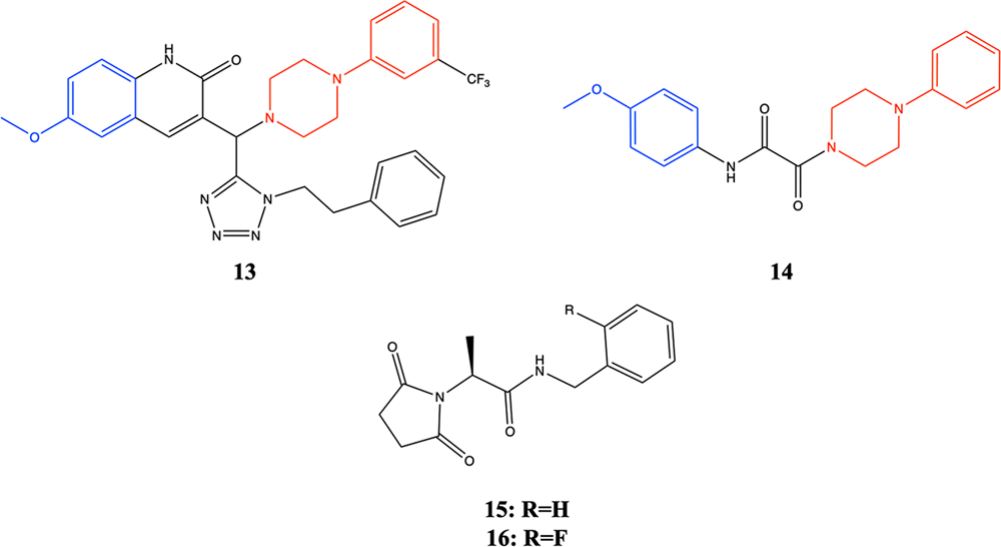
Structures of PAMs.

Kortagere and colleagues^155^ identified three selective PAMs of EAAT2, in which compound **13** (GT951, Figure 4) was considered the best mediator of glutamate excitotoxicity by interacting with the TM2, TM5, and TM8 (BS2, Figure 2) of EAAT2.^151,155^ In primary culture of neurons and astrocytes, compound **13** was neuroprotective improving the uptake of glutamate via EAAT2 in a noncompetitive mechanism.^151^ Glia incubated for 24 h with or without a compound similar to **13**, where the (trifluoromethyl) phenyl) piperazin motif is substituted with a cyclohexylpiperazin (GT949), revealed that EAAT2 expression is unaffected.^155^ Despite the high efficacy of **13**, it has very poor drug-like properties including high lipophilicity and poor aqueous solubility which limit its bioavailability *in vivo*. Next, an optimization campaign led to the design of **14** (GTS511, EC_50_ 3.8 ± 2.2 nM) with more favorable drug-like properties. Docking studies showed that **13** and **14** bind to BS2 with strong interactions forming hydrogen-bond, cation-π, and hydrophobic interactions, suggesting direct interactions with M86, L295, K299 (TM5), S465, and W472.^155,156^

The PAM **15** ((R)-AS-1, Figure 4) revealed favorable anticonvulsant and safety profiles, displaying a good permeability in the parallel artificial membrane permeability assay (PAMPA), an excellent metabolic stability in human liver microsomes (HLMs), no influence on CYP3A4/CYP2D6 activity, as well as no hepatotoxic properties in HepG2 cells. **15** protected mice significantly against seizures in acute animal models of seizures. **15** showed a potency of 25 ± 21 nM and efficacy of 174 ± 13% for glutamate uptake augmentation in cultured glia. Studies in cultured glial cells revealed that the addition of a fluorine atom in **16** (Figure 4) had a more potent effect (with an EC_50_ of 0.1 ± 0.3 nM) than what was observed for **15** (∼20 nM) with a similar efficacy of glutamate transport augmentation. Similarities between **13** and **15**–**16** included bioisosteric replacement of the 5,6-dihydropyridin-2(1H)-one ring with pyrrolidine-2,5-dione; the tetrazole moiety with an amide fragment, as well as exchange of phenylethyl substituent to benzyl. Molecular docking simulations on EAAT2 revealed that binding site of **15**–**16** coincides with the binding site of **13**. Molecular interactions stabilizing the bound form include a cation-π interaction between K299/R476 and the benzene ring of the compounds, strong hydrophobic interactions involving, e.g., M86, and hydrogen bonds between the pyrrolidine-2,5-dione ring and K90 and D238.^1570^

Because positive allosteric modulation is a fast direct process, it does not require synthesis and trafficking of new protein and will consequently have immediate acute effects and not rely on prophylactic pretreatments. PAMs of EAAT2 have a promising clinical potential by enhancing the removal of excessive glutamate in the synaptic cleft and preventing glutamate-mediated excitotoxicity. Future preclinical studies on PAMs in rodent models of neurodegeneration are needed to evaluate their efficacy.

## 5 EAAT2 Inhibitors

The ever-growing interest in understanding glutamate uptake and EAAT2 function led to the development of multiple EAAT2 inhibitors. Specifically, these compounds are of great interest to the development of PET radiotracers that could help to assess EAAT2 density in the living brain. In the following subsections, we describe the plethora of restricted glutamate analogues and aspartate derivatives that bind to EAAT2, constructing a structure–activity relationship to indicate which compounds have higher affinity and selectivity.

### 5.1 EAAT2 Inhibitors: Aspartate Analogues

**Figure 5:**
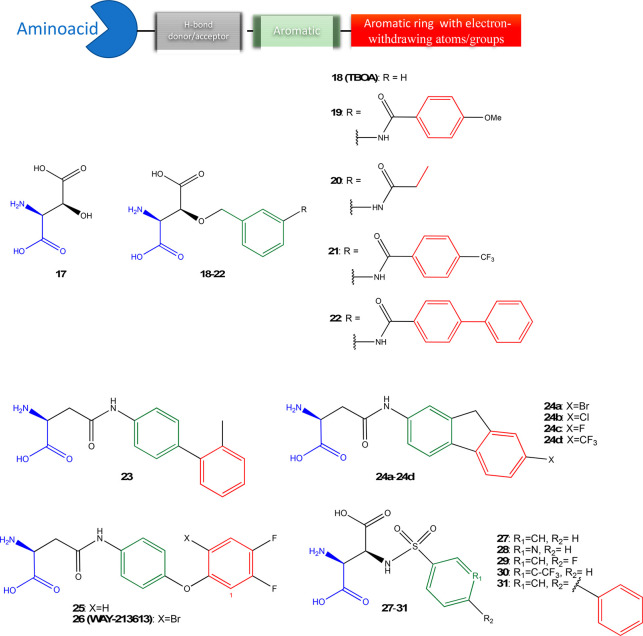
EAAT2 binders as aspartic acid analogues **17**–**31**.

l-glutamate and d- or l-aspartate bind to EAAT2.^157−159^ Back in the 1970s, Balcar and Johnston synthesized the aspartate analogue *threo*-3-hydroxy-l-aspartate (**17**, Figure 5), which has been described, for many years, as the most potent inhibitor of the EAAT’s (IC_50_ = 31 ± 5.8, Table 1).^160^**17** contains an amino and carboxylic acid (highlighted in blue in Figure 5), a hydrogen-bond donor/acceptor carboxylic acid (black), and an alcohol functional group (Figure 5). To understand the preferable conformation and isomeric forms to inhibit the glutamate uptake via EAATs, different stereoisomers of **17** were synthesized. A comparison between l-, d-, and dl-mixture demonstrated similar affinity to EAATs (IC_50_ = 3.2, 5.6, and 4.0 μM, respectively). Further studies established that **17** behaves as a EAAT’s substrate (i.e., uptake by the transporter)^36^ and lacks selectivity to inhibit glutamate uptake,^30^ binding also to NMDA receptors.^161^ These works have encouraged the development of selective aspartate analogues binding EAAT2. The hydroxyl group at the C3-position of **17** allows for exploring the structure–activity relationship of novel aspartate analogues and has provided strong inhibitors of the EAAT2 over the last decades.^162^

**Table 1:** Aspartate Analogues Inhibitors of EAAT2

	IC_50_ (μM)			Ratio		
Compounds	EAAT1	EAAT2	EAAT3	Ratio EAAT1 vs EAAT2	Ratio EAAT3 vs EAAT2	Ref
**17**	96 ± 13	31 ± 5.8	–	3.1	–	(163)
**18**	67 ± 7.5	5.5 ± 1	–	12.1	–	(163)
**19**	0.116	**0.059**	2.4	2.0	40.6	(170)
**20**	–	**1.2**	4.1	–	3.4	(170)
**21**	0.022	**0.017**	0.3	1.3	17.6	(170, 171)
**22**	–	**0.021**	0.034	–	1.6	(170)
**23**	2	**0.1**	10	20	100	(173)
**24a**	0.3	**0.2**	0.65	1.5	3.3	(173)
**24b**	0.15	**0.1**	0.2	1.5	2	(173)
**24c**	0.1	**0.08**	0.05	1.3	0.6	(173)
**24d**	3.8	**0.1**	2.4	38	24	(173)
**25**	2.9	**0.13**	14.5	22	111.5	(173)
**26**	5	**0.08**	3.8	62.5	47.5	(173)
**27**	0.8	**2.4**	1.2	0.3	0.5	(168)
**28**	18	**28**	13	0.6	0.5	(168)
**29**	1.6	**4.7**	2.0	0.3	0.4	(168)
**30**	100	**2.8**	150	35.7	53.5	(168)
**31**	2.3	**0.5**	20	4.6	40	(168)

**Figure 6:**
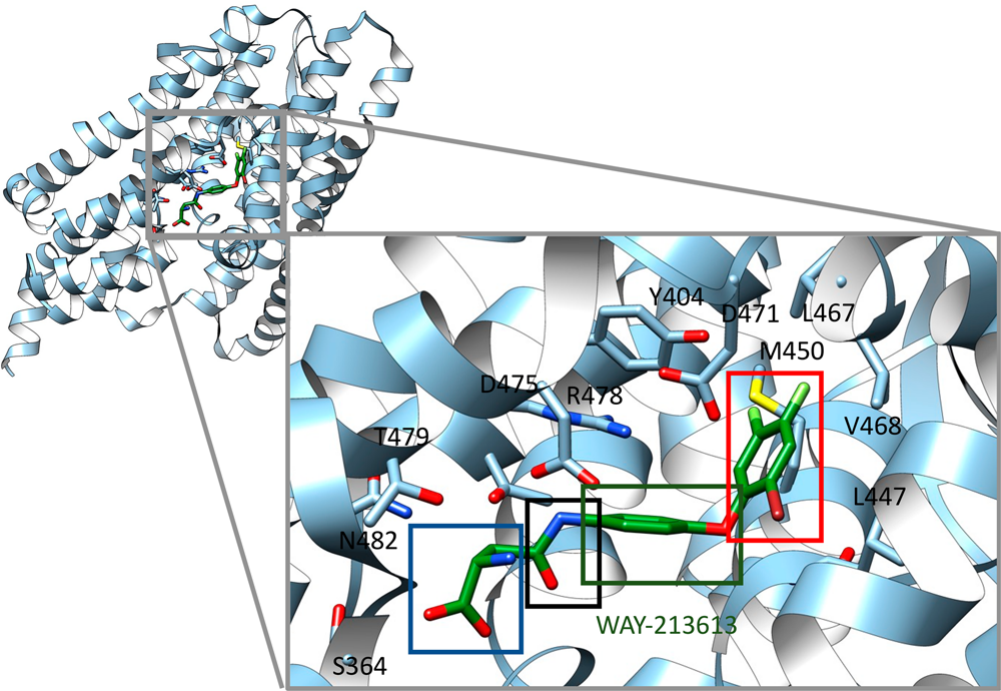
Binding mode of **26** (WAY213613, green) to EAAT2 (blue cartoon) (PDB ID code: 7XR6).

The dl-*threo*-β-benzyloxyaspartate (**18**, Figure 5) was synthesized by the addition of a aromatic substituent (highlighted in green in Figure 5) at the alcohol of **17**,^163^ with an attempt to avoid the substrate-like behavior of **17**.^36^ Indeed, **18** had a nonsubstrate inhibitor profile and selectivity for EAAT2 over GluRs (>100-fold).^163^ Currently, the **18** is commercially available and is widely applied in studies involving both physiological and pathophysiological roles of EAAT2.^164−166^ Yet, **18** lacked selectivity toward EAAT3, EAAT4, and EAAT5.^167^ The substitution of OCH_2_ group with amide group displayed a weak inhibitory activity at EAAT2 (IC_50_ ∼ 70 μM) while being inactive at EAAT1 and EAAT3 at concentrations up to 300 μM.^168^ Maintaining the ether group of **18**, the modification at the benzyloxy ring has been explored in the search of additional interactions in BP1 in order to improve EAAT2 selectivity.^169^ Indeed, slight variations in residues Leu467 and Val468 located around the tip of the aromatic group (Figure 6) are substituted with different sets of residues in EAAT1 and EAAT3, making the area of BP1 smaller, e.g., Leu467 (Figure 1) in EAAT2 is substituted with isoleucine in EAAT1 and EAAT3. Kato et al. using point mutations showed that these residues forming the cavities are closely related to the sensitivities of the EAAT subtypes to inhibitors.^51^ Indeed, the addition of an aromatic group (red in Figure 5) via amide bond (**19**, IC_50_ = 59 nM) increased the activity by ∼100-fold vs EAAT2. **19** was slightly more selective to EAAT2 than EAAT1(∼2-fold) and EAAT3 (and 40-fold).^170^ The substitution of the *para*-metoxyphenyl group with an aliphatic chain had a negative effect on activity (IC_50_ of **20** = 1200 nM, Figure 5, Table 1), suggesting that the molecule aromatic group favor the interaction with the EAAT2.^169^ The introduction of electron-withdrawing CF_3_ (**21**, also known as **TFB-TBOA**) instead of a methoxy group (**19**) increased IC_50_ by 3.5 times (IC_50_= 17 nM) and in a further comparison using COS-1 cells, showed a 17-fold selectivity vs EAAT3, but not over EAAT1.^170,171^ Leuenberger et al. have shown that the relative stereochemistry of **21** is crucial for high inhibitory activity, with the 2,3-*syn* (*threo*) isomers being clearly more active than the 2,3-*anti* (*erythro*) isomers.^172^ The substitution of the CF_3_ group with an aryl ring (**22**, Figure 4, Table 1) decreased the affinity to EAAT2 by 2 times as the bulky ring might not fit in the BS1. Of note, analogues **17**–**22** are unlikely to cross the BBB,^170^ limiting their application *in vivo*.

To improve physicochemical properties and aiming for a molecule that may penetrate the BBB, a series of aspartamide and 2,3-diaminopropionic-acid were developed.^173^ Aspartamide molecules are composed of only one free carboxylic acid, which decreases the number of hydrogen bond acceptors and charge favoring BBB penetration. It was first observed that the biphenyl in compound **23** (Figure 5) is essential for inhibitory activity.^173^ In addition, increasing the rigidity of **23** into a fluorene (Figure 5) and adding an halogen atom resulted in similar affinity (IC_50_ = 0.2 μM (**24a**, Br) > 0.1 μM (**24b**, Cl) = 0.1 μM (**24d**, CF_3_) > 0.07 μM (**24c**, F)), with slight improvement with the use of electron-withdrawing fluorine but losing selectivity versus EAAT3. The increased affinity obtained with perturbations in the biaryl bond of **23** led to the development of ether derivatives. The ether derivative **25** (Figure 5) bearing two fluorine atoms maintained the same affinity as **24c** but owns higher EAAT2 selectivity.

Introducing a bromide atom in **26** (Figure 5) resulted in the best-in class compound with IC_50_ of 0.08 μM toward EAAT2 and 59- and 45-fold higher selectivity versus EAAT1 and EAAT3, respectively (Table 1). Noteworthy, **26**, which is commercially available as WAY213613, remains the most selective and potent EAAT2 inhibitor to date.^173,174^ Cryo-EM analysis has recently shown that WAY213613 inhibits glutamate uptake via EAAT2 by either interaction at the glutamate-binding site and sterically preventing HP2 loop movement (Figure 6).^51^ The l-asparagine (Figure 6, blue and black squares) and 4-(2-bromo-4,5-difluorophenoxy) phenyl (Figure 6, green and red squares) moieties of **26** played distinct roles in the EAAT2 inhibition, by competing with the glutamate binding and sterically preventing the HP2 loop gating suspending the transport cycle of glutamate, respectively.

Hansen et al. synthesized thirty-two β-aspartate analogues (Figure 5).^168^ The authors proposed a key role for the β-position of aspartate in the glutamate uptake inhibition. Interestingly, the β-sulfonamide scaffold (**27**) has inhibitory activity in the low micromolar range (IC_50_ = 2.4 μM) but lacks selectivity to EAAT2. Of note, the l-*threo* conformation of **27** has an IC_50_ of 2.4 μM, while the l-*erythro* is inactive (IC_50_ = 200 μM). Thus, l-*threo***27** became the main scaffold to develop further EAAT2 selective inhibitors. Methylation of the sulfonamido group or rotational restriction of the phenyl ring by introduction of two *ortho*-chloro atoms decreased the inhibitory EAAT potency significantly. Heteroaromatic analogues **28** (Figure 5) demonstrated considerable loss of selectivity. The 4-fluoro analogue **29** demonstrated similar inhibitory potency at EAAT2 (IC_50_ 4.7 μM) as **27**; however, increasing the number of fluorine atoms decreased the inhibitory potency of the analogues toward EAAT2. The *meta*-CF_3_ substituted analogue **30** is a potent inhibitor of EAAT2 and possesses high selectivity toward EAAT1 and EAAT3.^168^ As anticipated, the EAAT2 selectivity might be also reached by the addition of an aromatic ring, indeed, **31** bearing a phenyl ring in para position displayed high inhibitory potency at EAAT1 and EAAT2 (IC_50_ values of 2.3 and 0.50 μM, respectively) and 10–40-fold lower potency at EAAT3.

### 5.2 EAAT2 Inhibitors: Glutamate Analogues

**Figure 7:**
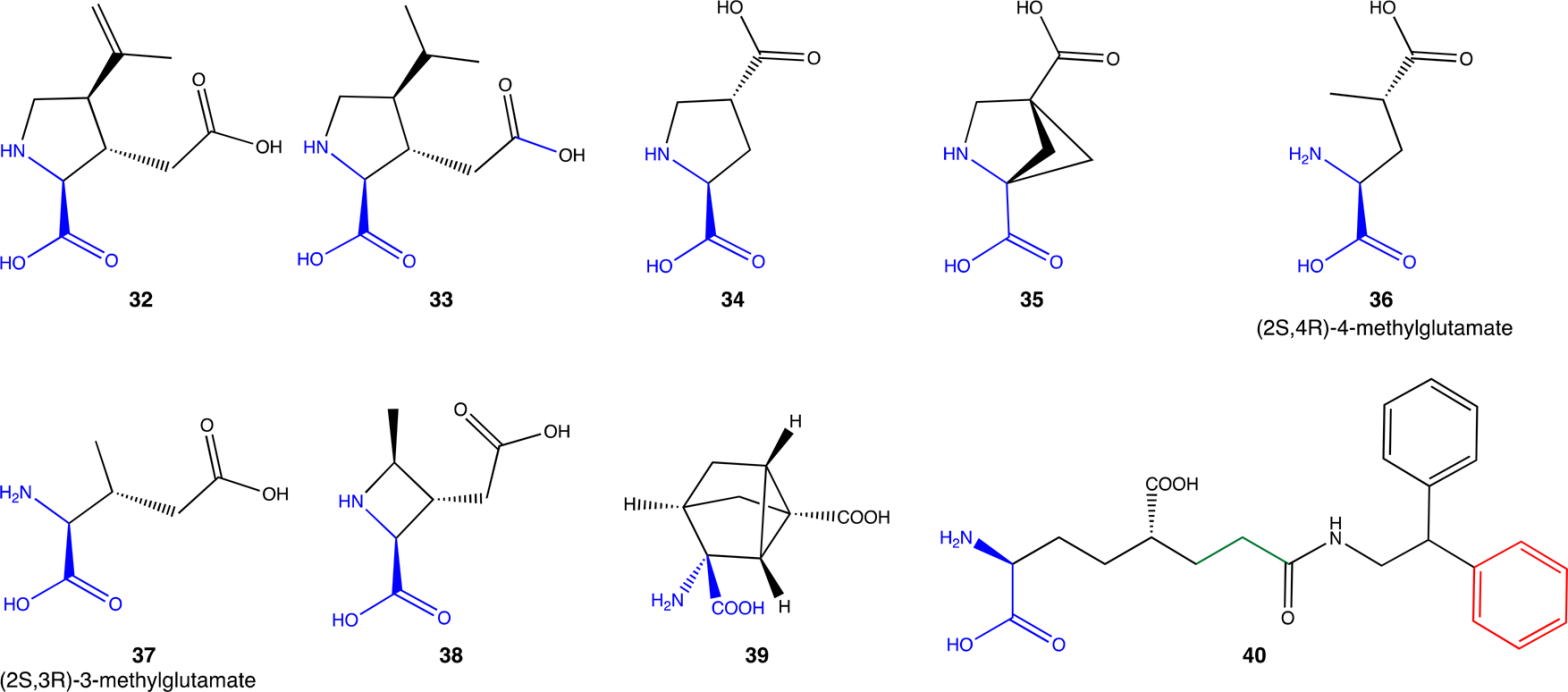
EAAT2 inhibitors as glutamate analogues **32**–**39**.

To date, many conformationally restricted glutamate analogues have been synthesized to help shed light on the characterization of glutamate receptors and EAAT’s (Figure 7). Concomitantly to the development of aspartate derivatives in the beginning of the 1970s, the glutamate analogue **32** (also known as kainic acid, Figure 7) was shown to inhibit EAAT2 (*K*_i_ = 59 ± 18 μM, Table 2).^157,175^ Specific binding in the rat brain using [^3^H]**32** was observed in the striatum, hippocampus, cerebral cortex, hypothalamus, and cerebellum.^176^ The analogue **33** (also known as DHK, Figure 6) is twice as potent as **32**(175) and highly selective to EAAT2 vs glutamate receptors.^157^ Further analyses of **32** and **33** mechanisms of inhibition demonstrated that both compounds are competitive antagonists of EAAT2-mediated glutamate uptake.^157^ Interestingly, **34** (also known as PDC) has a inhibitory activity in the low micromolar range (*K*_i_ = 8 μM) but with lower selectivity toward EAAT2 (EAAT1 = 10-fold, EATT3 = 8-fold).^157^ In mice primary cortical cocultures of neurons and astrocytes, treatment with **34** triggered an elevation of extracellular glutamate concentration, induced neuronal calcium influx, and NMDA receptor (NMDAR) mediated-neuronal death without having any direct agonist activity on NMDARs.^177^ Although compound **35** exhibited a high degree of overlap with **34**, it was proved to be an excellent substrate of EAAT2.^178^

**Table 2:** Glutamate Analogues Inhibitors of the Excitatory Amino Acid Transporter 2

	*K*_i_ (μM)			Ratio		
Compound	EAAT1	EAAT2	EAAT3	Ratio EAAT2 vs EAAT1	Ratio EAAT2 vs EAAT3	Ref
**32**	>3000	59 ± 18	>3000	>100	>100	(157)
**33**	>3000	23 ± 6	>3000	>100	>100	(157)
**34**	79 ± 7	8 ± 2	61 ± 14	9.9	7.6	(157)
**40**	1000–3000	95	3000	10.5–31.6	31.6	(181)

Vandenberg et al. investigated a series of methyl derivatives at 3- or 4-position of glutamate on EAAT2 by modeling the conformations of the methylglutamate derivatives to the restricted conformation of **32**, which inhibited the transport of glutamate by blocking the transporter, and found that 4-methylglutamate derivative ((2*S*,4*R*)-4-methylglutamate) **36** were transported by EAAT1 but potently blocked glutamate transport via EAAT2.^179^ Interestingly, the epimer (2*S*,4*S*) of **36** was inactive either as a blocker or as a substrate for both EAAT1 and EAAT2.^179^ The *threo*-3-methylglutamate derivative **37** (Figure 6) is a competitive blocker of EAAT2, while the *erythro*-isomer did not inhibit the transport glutamate. This highlights the importance of stereoisomerism at positions 3 and 4 in defining a nonsubstrate inhibitor profile.^179^

Conformationally constrained azetidine **38** was identified as an inhibitor and showed selectivity for the EAAT2 subtype (versus EAAT1) and potency on EAAT2 equiv to **33**.^179^ The selectivity toward the EAAT2 subtype was lost by elimination of a the 4-alkyl substituent.^179^ The similarities on **32**–**38** suggest that all three molecules might bind to the same or closely related sites on EAAT2 near the extracellular surface of the protein. Pharmacophores have also assisted in the identification of the chemical properties and geometry that favor a nonsubstrate EAAT2 inhibitor.^178^ Based on this, Dunlop et al. developed compound **39** (WAY-855) which contains a glutamate backbone in a six-member heptane dicarboxylate ring.^180^**39** has 45- and 11-fold selectivity over EAAT1 and EAAT3, respectively, and behaves as a nonsubstrate inhibitor of EAAT2 (IC_50_ = 2.2 μM). Thus, conformational restriction and bulkiness is a key characteristic to obtain a selective nonsubstrate inhibitor of EAAT2.^180^ Application of **39** to EAAT2-injected oocytes blocked the inward current generated by glutamate. Results from the cell line uptake studies indicate selectivity for EAAT2 inhibition over EAAT3 and EAAT1 of 11- and 45-fold, respectively

Glutamate analogues with different functionalities in C4-position showed similar findings.^181^ The bulky substituent on the amide nitrogen of compound **40** resulted in intermediate affinity (*K*_i_ = 95 μM, Table 2) to EAAT2 and ∼31-fold selectivity over EAAT1 and EAAT3.^181^

## 6 Developing a PET Radiotracer Targeting EAAT2

PET radiotracers are unique tools for imaging receptors and transporters in the living brain.^182^ Nevertheless, the design of novel PET radiotracers comprises a few parameters that must be carefully considered, such as the selection of a molecule with high affinity and selectivity to the target. In section 5 we discussed the library of known EAAT2 inhibitors, in which the compound **24c** (commercially available as WAY213613) represents the most promising molecule for designing a PET radiotracer with high affinity and selectivity to EAAT2.

Importantly, the affinity required for a radiotracer is highly dependent on *B*_max_ , the concentration of the target in the region of interest (ROI). (For a more detailed discussion on the designing and testing of radiotracers, see ref (183).) EAAT2 *B*_max_ in the hippocampus of an adult rat has a value of ∼199 nM.^46^ In keeping with this, the desired inhibitory affinity should be approximately 20 nM or less to detect EAAT2 in the hippocampus. For imaging brain receptors, the minimum ratio of the *B*_max_/*K*_d_ should be at least 10, in order to achieve an acceptable signal in the *in vivo* PET imaging analysis.^184^ Nevertheless, an intermediate affinity (e.g., 20 nM < *K*_d_ > 100 nM) may be tolerated if selectivity is high.^184^ The compound **24c** is 59- and 45-fold higher selective vs EAAT1 and EAAT3 with affinity toward EAAT2 of 80 nM. Molar activity of the PET radiotracer at the time of injection should also be considered (for a more detailed discussion, see ref (185)).

**Figure 8:**
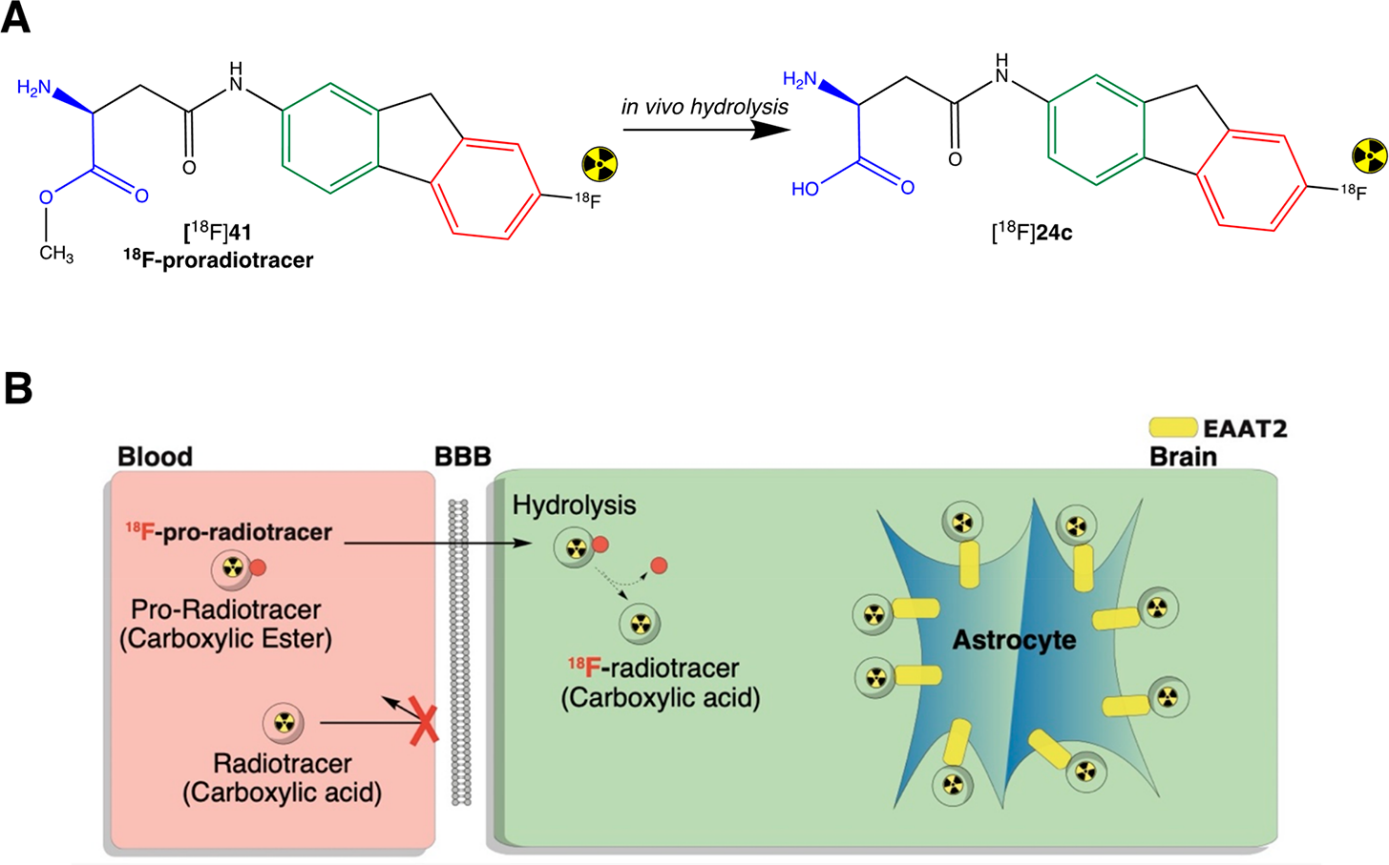
Pro-radiotracer approach to increase the BBB permeability for EAAT2 inhibitors. (A) The EAAT2 specific inhibitor developed **24c** by Greenfield et al. possesses a free carboxylic acid–a disadvantage for brain penetration.^173^ A pro-radiotracer approach may be applied to increase BBB permeability. The ^18^F-pro-radiotracer [^18^F]**41** was developed following the addition of a methyl ester in the C-terminal of the EAAT2 inhibitor. After *in vivo* administration, the [^18^F]**41** is hydrolyzed in the brain, producing the EAAT2-specific ^18^F-radiotracer [^18^F]**24c** inside the brain. (B) Schematic mechanism of a brain pro-radiotracer.

Of note, in addition to the compound affinity and selectivity, accessibility to the ROI must be considered. PET radiotracers are commonly administered intravenously, thus, it seems reasonable to estimate whether the selected molecule is likely to “access the brain”, i.e., cross the BBB, and bind to the EAAT2. Wager and colleagues proposed the CNS multiparameter optimization desirability tool, in which a few physicochemical parameters must be evaluated to increase the chances of a molecule to cross the BBB.^186^ In this context, the free carboxylic acid functionality of **24c** may impact the molecule permeability through the BBB due to its high polarity and negative charge at physiological pH.^187^ To overcome this possible limitation and increase BBB permeability, different approaches could be applied, such as replacing the carboxylic acid for tetrazole isostere.^188^ In addition, the radiosynthesis of the first EAAT2-selective radiotracer highlighted that replacing the free carboxylic acid for a carboxylic ester increased the BBB permeability without compromising EAAT2 affinity and selectivity (a clinical trial is ongoing: NCT05374278). This strategy in which the radiotracer mimics a pro-drug could be identified as the “pro-radiotracer approach”. Indeed, dynamic PET imaging of 6-[^18^F]bromo-7-(2-fluoroethyl) purine ([^18^F]**41**, Figure 8), a PET “pro-radiotracer”, demonstrated good brain uptake and fast metabolic conversion, suggesting that this could be, indeed, a promising strategy to deliver PET radiotracers selective for targets in the brain.^189^ Another possibility to develop a PET radiotracer could be to radiolabel with carbon-11 at the methoxy group **13**–**14** PAMs or with fluorine-18 compounds **13** and **16** as these compounds have pharmacokinetic profiles suitable to image EAAT2 in the brain.

## 7 Concluding Remarks

EAAT2 plays a key role in maintaining glutamatergic homeostasis in the mammalian brain. Human and rodent data support the involvement of EAAT2 loss/dysfunction in neurodegenerative diseases. These findings led to the development of innovative PAMs and translational activators as potential therapeutic agents to prevent glutamate excitotoxicity via EAAT2. Nevertheless, the appropriate time point for therapeutic intervention, in which EAAT2-targeting drugs might be effective to prevent neurodegeneration, is still unknown. Therefore, it is crucial to find noninvasive ways to improve our knowledge on EAAT2 function/expression in the living brain. In this context, neuroimaging techniques such as PET hold promise, but no PET radiotracer with high selectivity and affinity to EAAT2 has been available for clinical research so far. We believe that the availability of the cryo-EM structures of EAAT2 will offer the opportunity to initiate a structural-based drug design campaign to facilitate the development and screening of high-affinity PAMs able to modulate EAAT2 activity while using them as imaging tools to detect change in EAAT2 density in neurodegenerative diseases.

## Abbreviations Used

AβOs: amyloid-β oligomers
AT(N): amyloid, tau and neurodegeneration
BP: binding pocket
CN: calcineurin
CREB: cAMP-response element binding protein
cryo-EM: cryogenic electron microscopy
CYP46: cholesterol 24S-hydroxylase
EAAC1: excitatory amino acid carrier 1
EAAT: excitatory amino acid transporter
EAAT2-e9: excitatory amino acid transporter 2 skipped exon-9
fALS: familial amyotrophic lateral sclerosis
GFAP: glial fibrillary acidic protein
GLT-1: glutamate transporter 1
HD: Huntington’s disease
HP: hairpin
LRRK2: leucine-rich repeat kinase 2
NFAT: nuclear factor activated T-cells
ROI: region of interest
SLC1: solute carrier 1
SN: system N transporter
SOD1: superoxide dismutase 1
TM: transmembrane
vGluT: vesicular glutamate transporter
