# Correction to “A Medicinal Chemistry Perspective on Excitatory Amino Acid Transporter 2 Dysfunction in Neurodegenerative Diseases”

**DOI:** 10.1021/acs.jmedchem.3c00353

**Published:** 2023-03-14

**Authors:** Igor C. Fontana, Débora G. Souza, Diogo O. Souza, Antony Gee, Eduardo R. Zimmer, Salvatore Bongarzone

Page 2335. This Perspective was published with an incorrect absolute configuration of compounds **13** and **15**−**16** in Figure 4. The corrected figure is shown below.

**Figure 4:**
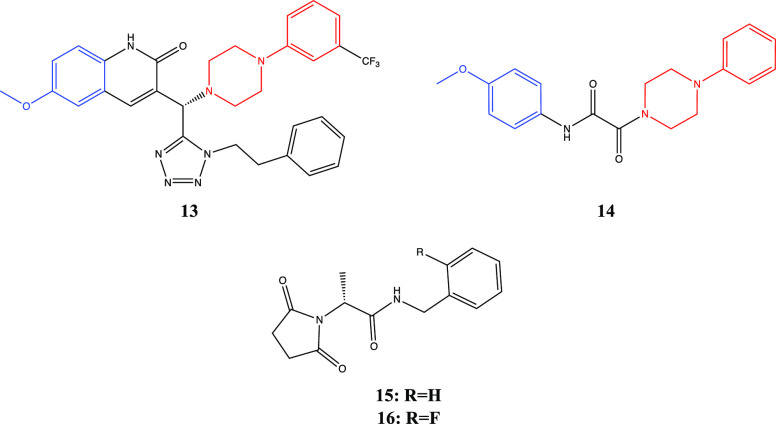
Structures of PAMs.

